# Comparative Evaluation of Scleral Lens Treatment in Ocular Surface Diseases: A Prospective Study

**DOI:** 10.1155/joph/1921583

**Published:** 2026-05-23

**Authors:** Yirui Zhu, Jiangxiong Cai, Li Su, Jilian Dong, Jie Zhou, Haohan Zheng, Yujie Mou, Xiuming Jin

**Affiliations:** ^1^ Eye Center, The Second Affiliated Hospital, School of Medicine, Zhejiang University, Hangzhou, Zhejiang, China, zju.edu.cn; ^2^ Zhejiang Provincial Key Laboratory of Ophthalmology, Hangzhou, Zhejiang, China; ^3^ Department of Ophthalmology, Wenling Hospital Affiliated to Wenzhou Medical University (The First People’s Hospital of Wenling), Taizhou, Zhejiang, China; ^4^ School of Public Health, China Medical University, Shenyang, China, cmu.edu.tw; ^5^ Department of Ophthalmology, Songyang Hospital of Traditional Chinese Medicine, Lishui, China

**Keywords:** midday fogging, ocular surface disease, scleral lens, tear reservoir, visual rehabilitation

## Abstract

**Purpose:**

This study evaluated the clinical efficacy and patient‐reported outcomes of scleral lens wear across different ocular surface diseases, providing evidence for individualized fitting and management strategies.

**Methods:**

Patients with keratoconus, ocular surface trauma, postkeratoplasty, and dry eye were fitted with scleral lenses using OCT‐guided customization. All participants underwent baseline examinations before and after lens wear, including best‐corrected visual acuity (BCVA), intraocular pressure (IOP), central corneal thickness (CCT), and Ocular Surface Disease Index (OSDI) assessment. Patient‐reported outcomes, including quality of life, comfort, visual clarity, handling, cleanliness, and satisfaction, were collected via a standardized questionnaire.

**Results:**

BCVA improved significantly in all groups after scleral lens wear, with the postkeratoplasty group showing the least improvement. OSDI scores decreased significantly across the groups. CCT increased after lens wear, indicating mild corneal thickening. The postkeratoplasty group exhibited significantly greater tear reservoir thickness at the superior and superonasal locations compared to the keratoconus and dry eye disease groups. Subjective ratings of handling, cleanliness, and satisfaction revealed no significant differences across the four groups. However, the trauma group reported less improvement in quality of life, and the postkeratoplasty group experienced lower comfort levels compared to the keratoconus group. Overall, 51.3% of patients reported experiencing midday fogging.

**Conclusions:**

Scleral lenses offer effective visual rehabilitation and symptom relief across diverse ocular surface diseases, with consistently high levels of patient‐reported satisfaction. Future research should focus on individualized fitting strategies, address challenges such as midday fogging, and evaluate long‐term safety and efficacy to guide wider clinical applications.

**Trial Registration:** ClinicalTrials.gov identifier: NCT06555367

## 1. Introduction

Scleral lenses are large‐diameter gas‐permeable contact lenses that vault over the cornea and rest on the sclera, creating a tear reservoir between the posterior lens surface and the cornea. Scleral lenses have been the subject of substantial material and design innovations, making them a powerful tool in both visual rehabilitation and ocular surface protection [1]. Their unique ability to neutralize corneal irregularities, maintain a stable refractive surface, and provide the eyes with continuous hydration makes them particularly useful for managing a wide range of complex ocular disorders.

Recent studies have confirmed the high success and patient satisfaction rates of scleral lenses for individuals with refractory ocular surface disease [2]. Given the increasing availability of high oxygen‐permeable materials and advanced fitting technologies, the clinical applications of these lenses have expanded beyond traditional indications, such as keratoconus, to include postsurgical corneas, ocular trauma, limbal stem cell deficiency, and severe dry eye disease [3, 4]. For patients with keratoconus, scleral lenses are a nonsurgical option for achieving optical regularization when spectacles and soft lenses fail, especially in advanced or asymmetric cases [5, 6]. In postkeratoplasty eyes, scleral lenses can effectively manage high irregular astigmatism and anisometropia, providing a safer and more stable alternative to corneal rigid gas‐permeable lenses [7]. In eyes with irregular astigmatism secondary to trauma or surgical intervention, the scleral vault allows complete corneal clearance, protecting against further mechanical insult and supporting epithelial healing [8]. Moreover, the reservoir of tears maintained between the lens and the cornea has been demonstrated to have clinical benefits in patients with severe dry eye disease, ocular graft‐versus‐host disease (GVHD), Stevens–Johnson syndrome (SJS), and limbal stem cell deficiency by promoting continuous hydration and improving epithelial integrity [9–12].

Despite increasing interest in scleral lens technology, most existing studies have concentrated on a single underlying condition, most often keratoconus, while lacking comparative evaluations across different etiologies. This limitation reduces the generalizability of existing findings and impedes the development of condition‐specific fitting strategies. For instance, eyes with postkeratoplasty irregularities or ocular trauma may have markedly different corneoscleral geometries, tear film, and ocular surface sensitivity from keratoconus or dry eye, potentially requiring distinct fitting approaches and clinical expectations [13].

Furthermore, while numerous studies have reported that scleral lenses improve visual acuity or ocular surface comfort, few have comprehensively integrated both objective clinical outcomes and patient‐reported measures, such as perceived comfort, vision clarity, ease of lens handling, and quality‐of‐life impact [14]. These subjective metrics have increasingly been recognized as critical indicators of fitting success and overall treatment efficacy, particularly for long‐term scleral lens tolerance and patient adherence.

Therefore, this study evaluates the clinical performance of scleral lenses across multiple ocular surface diseases by integrating objective clinical measurements with subjective patient‐reported outcomes. By conducting a cross‐etiology comparison, we highlight both consistent improvements in visual acuity and ocular surface symptoms and disease‐specific variations in corneal response and patient‐reported quality of life, thereby providing evidence‐based guidance for personalized scleral lens fitting in clinical practice.

## 2. Patients and Methods

A prospective, open‐label clinical experiment was conducted for this study. The experiment was authorized by the hospital’s institutional review board and recorded in the Clinical Trial Registry (NCT06555367). The research was conducted from October 2023 to December 2024 at the Eye Clinic of Zhejiang University’s Second Affiliated Hospital. The participants provided their written informed consent before taking part in the study. This study was approved by the Institutional Review Board of the Affiliated Second Hospital of Zhejiang University.

### 2.1. Subjects

Patients were categorized into four groups based on the disease type: the keratoconus group, the poskeratoplasty group, the trauma group, and the dry eye disease group. The participants were patients fitted with scleral lenses who visited the eye clinic and met the following criteria: (1) able to tolerate scleral lens wear without age and gender restriction (2) and had irregular corneal astigmatism or severe dry eye, including those with keratoconus, postkeratoplasty, and severe irregular corneal astigmatism caused by trauma.

Exclusions were based on the following criteria: (1) any ocular disease or surgery that contraindicates contact lens wear, such as ocular infection, allergy, intraocular inflammation, or trauma; (2) corneal endothelial cell density < 1000 cells/mm^2^; (3) poor compliance with lens wear instructions and follow‐up; (4) inadequate hygiene conditions for safe contact lens use; (5) pregnancy or lactation; (6) allergy to contact lenses or lens care solutions; (7) occupational or environmental conditions unsuitable for contact lens wear; and (8) any other ocular condition that could affect the ocular surface.

### 2.2. Prefitting Examinations

All patients underwent comprehensive prefitting evaluations, including a slit‐lamp biomicroscopy of the anterior segment, uncorrected visual acuity (UCVA), best‐corrected visual acuity (BCVA), intraocular pressure (IOP) measurement, fundus examination, central corneal thickness (CCT), anterior segment analysis using the Pentacam HR system, swept‐source anterior segment optical coherence tomography (AS‐OCT, Intalight), corneal endothelial cell count, and Ocular Surface Disease Index (OSDI) questionnaire assessment.

The AS‐OCT scleral lens mode was selected for all examinations. The device’s built‐in software was then used to automatically calculate the 360° sagittal depth, average sagittal depth, white‐to‐white (WTW) distance, chord length (defined as the linear distance parallel to the scleral spur intersecting with the sclera), and scleral angle (defined as the angle between the tangent line at the intersection point of the sclera and the chord parallel to the scleral spur). Postlens fitting assessments were performed using the same protocol. Postlens tear reservoir thickness was measured and recorded at the central, superior, inferior, nasal, temporal, superonasal, and inferotemporal locations.

### 2.3. Selection of Scleral Trial Lens

Patients with ocular surface diseases were fitted with trial scleral lenses of the Epicon A model (Capricornia, Australia) made from Boston XO2 material. These lenses have ultra‐high oxygen permeability and possess an oxygen permeability coefficient (Dk) of 141. A 3D simulated anterior segment image with a 15‐mm scan range was generated using AS‐OCT. The diameter of the trial lens was selected based on the WTW distance: for WTW ≤ 12 mm, a 14.5‐mm diameter lens was chosen, and for WTW > 12 mm, a 16.0‐mm diameter lens was selected. The device software automatically generated a horizontal chord line (13.5 mm or 15.0 mm in length) parallel to the iris and intersecting the sclera in a direction parallel to the scleral spur. A vertical line was then drawn from the corneal apex to measure the sagittal depth of the anterior segment. The initial trial lens sagittal depth was determined by adding 300 μm to the measured sagittal depth at a chord length of 13.5 mm or 15.0 mm.

### 2.4. Postscleral Lens Fitting Assessment

BCVA was assessed after 4 hours of continuous scleral lens wear and following completion of the OSDI questionnaire. After the four‐hour wear period, AS‐OCT was performed to measure and record CCT as well as the postlens tear reservoir thickness at the central and various peripheral locations with the lens in situ.

A customized questionnaire was designed to evaluate the patients’ postwear experience with scleral lenses (see Supporting Figure 1). The questionnaire’s subjective items included perceived improvements in quality of life, wearing comfort, visual clarity, lens handling, and lens cleanliness. Each item was scored on a 0–5 scale, with 0 indicating “unacceptable or very poor” and 5 indicating “excellent.” Overall satisfaction and overall evaluation were graded on a 5‐point scale, with 1 denoting “very satisfied” and 5 denoting “very dissatisfied.” Willingness to continue using scleral lenses was assessed on a 2‐point scale, with 1 representing “willing to continue wear” and 2 representing “unwilling to continue wear.”

### 2.5. Statistical Analysis

Continuous variables were expressed as mean ± standard deviation (SD). In this study, all continuous data followed a normal distribution and analyzed using the paired *t*‐test for parametric comparison. Non‐normally distributed data were compared using the Kruskal–Wallis test followed by Dunn’s test with Bonferroni‐corrected *p* values for pairwise comparisons. All statistical analyses were performed using SPSS Statistics 27 (SPSS, IBM Corporation, Chicago, IL, USA). *p* values < 0.05 were regarded as statistically significant for all two‐sided statistical tests.

## 3. Results

A total of 84 patients (104 eyes) were included in this study. The baseline demographic and clinical characteristics of the subjects are presented in Table 1. Postfitting BCVA, IOP, OSDI, and corneal thickness at the thinnest point for each group are shown in Table 2.

**TABLE 1:** Analysis of general scleral lens characteristics before lens wear.

Group	Keratoconus	Postkeratoplasty	Trauma	Dry eye
Gender				
Male	24 (82.8%)	22 (88.00%)	15 (100%)	5 (33.3%)
Female	5 (17.2%)	3 (12.00%)	0 (0.00%)	10 (66.7%)
Number of eyes	38	25	15	26
Age (years) (±SD)	25.00 ± 5.19	35.32 ± 15.56	30.67 ± 15.90	31.96 ± 11.01
Intraocular pressure	13.58 ± 3.09	12.96 ± 3.16	14.27 ± 2.91	14.34 ± 3.12
OSDI	43.74 ± 7.61	44.18 ± 10.76	38.33 ± 9.54	53.04 ± 7.66
Uncorrected visual acuity	0.23 ± 0.16	0.12 ± 0.11	0.25 ± 0.49	0.55 ± 0.26
Corrected visual acuity	0.52 ± 0.14	0.24 ± 0.19	0.33 ± 0.15	0.76 ± 0.23
K1 (D)	49.54 ± 6.89	46.00 ± 8.94	41.11 ± 2.84	41.21 ± 3.53
K2 (D)	68.69 ± 93.04	55.25 ± 9.99	49.55 ± 5.58	43.45 ± 3.03
Km (D)	51.52 ± 6.85	50.05 ± 9.28	44.68 ± 3.05	42.29 ± 3.20
Kmax (D)	63.08 ± 10.64	62.47 ± 10.78	55.31 ± 6.60	46.83 ± 2.71
Corneal thickness at the thinnest point (μm)	491.08 ± 47.42	562.79 ± 115.99	585.16 ± 57.63	501.87 ± 65.51
Corneal diameter (mm)	11.86 ± 0.39	11.86 ± 0.44	11.47 ± 0.28	11.54 ± 0.34

**TABLE 2:** Analysis of general scleral lens characteristics after lens wear.

Group	Visual acuity after lens wear	OSDI	Corneal thickness at the thinnest point (μm)	Intraocular pressure
Keratoconus	0.94 ± 0.16	19.32 ± 6.32	501.70 ± 42.05	14.52 ± 2.70
Postekeratoplasty	0.57 ± 0.36	24.82 ± 7.60	586.64 ± 97.78	14.28 ± 2.94
Trauma	0.77 ± 0.20	24.81 ± 9.87	596.85 ± 58.82	15.97 ± 2.98
Dry eye	1.03 ± 0.15	26.98 ± 6.34	509.49 ± 67.68	15.80 ± 3.11

All groups demonstrated significant improvement in visual acuity following scleral lens wear. In the keratoconus group, BCVA improved from 0.52 ± 0.14 to 0.94 ± 0.16; in the postkeratoplasty group, from 0.24 ± 0.19 to 0.57 ± 0.36; in the ocular trauma group, from 0.33 ± 0.15 to 0.77 ± 0.20; and in the dry eye disease group, from 0.76 ± 0.23 to 1.03 ± 0.15. These differences were statistically significant (*p* < 0.05, Figure 1(a)).

FIGURE 1(a) Best corrected visual acuity (BCVA) significantly improved after lens wear in all groups. (b) Ocular surface disease index (OSDI) scores markedly decreased following lens wear, indicating relief of ocular surface symptoms. (c) Intraocular pressure (IOP) changes before and after scleral lens wear across groups. (d) Central corneal thickness (CCT) exhibited a significant increase after lens wear, suggesting mild corneal edema or postlens tear accumulation (^∗∗^ *p* < 0.01 and ^∗∗∗^ *p* < 0.001).

**Figure:**
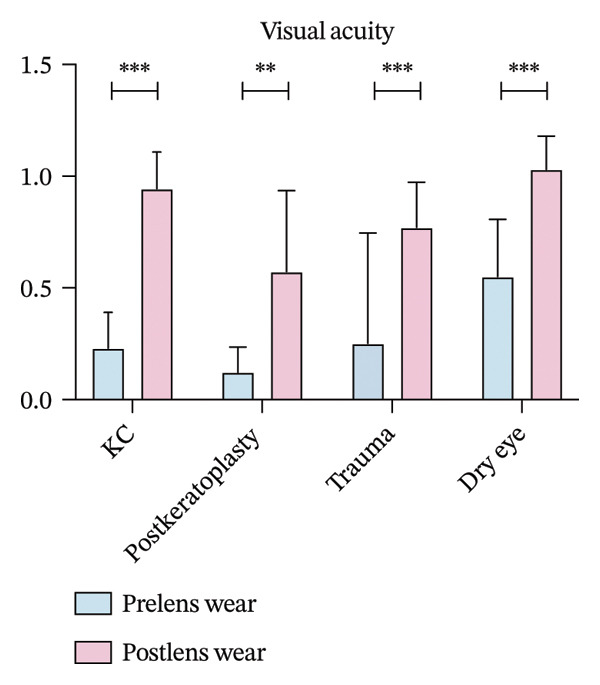
(a)

**Figure:**
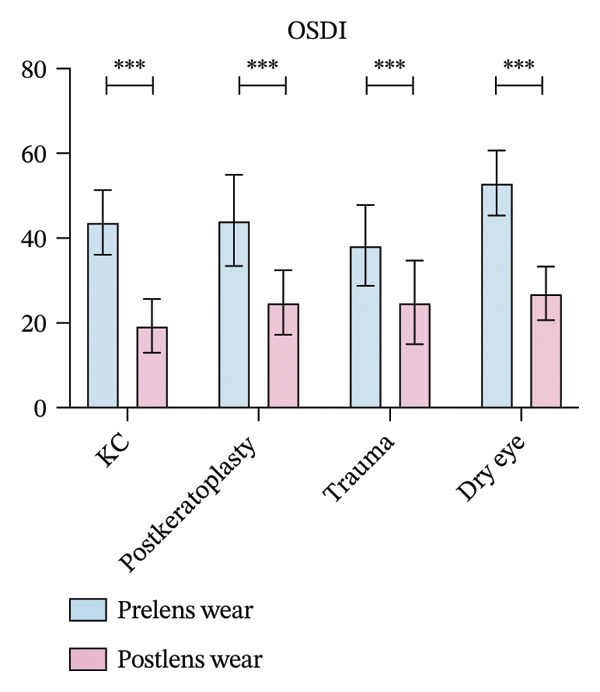
(b)

**Figure:**
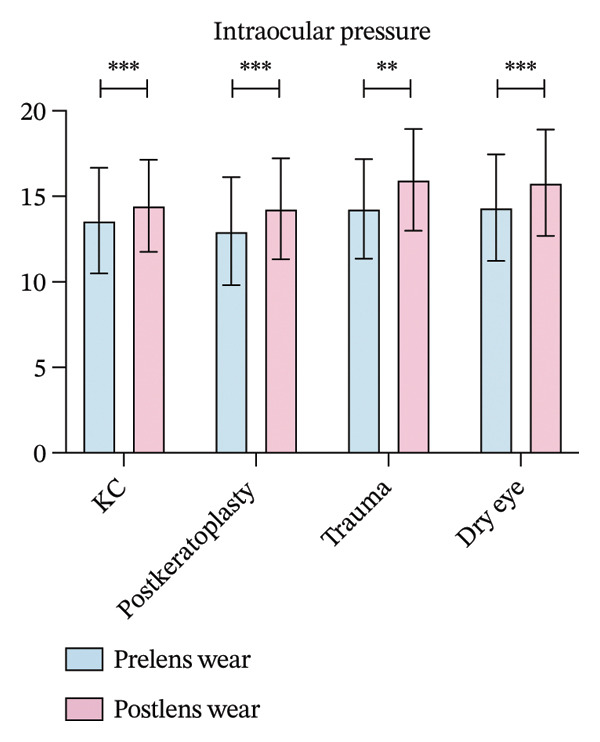
(c)

**Figure:**
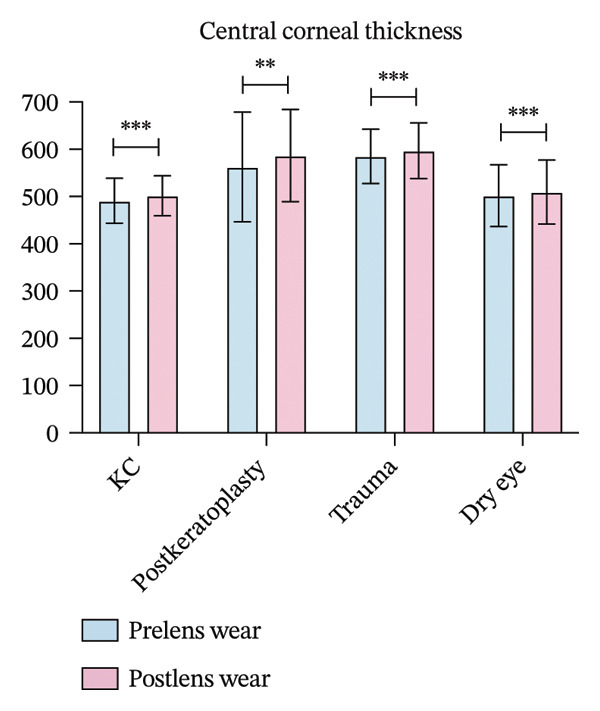
(d)

The OSDI scores significantly decreased in the four groups after scleral lens wear. In the keratoconus group, the OSDI score was significantly reduced from 43.74 ± 7.61 to 19.32 ± 6.32 (*p* < 0.001). Moreover, in the postkeratoplasty group, the score decreased from 44.18 ± 10.76 to 24.82 ± 7.60 (*p* < 0.01). In the ocular trauma group, the OSDI score significantly decreased from 38.33 ± 9.54 before scleral lens wear to 24.81 ± 9.87 after wear (*p* < 0.001). In the dry eye disease group, the score decreased from 53.04 ± 7.66 to 26.98 ± 6.34 (*p* < 0.001, Figure 1(b)).

IOP significantly increased in the four groups after 4 h of scleral lens wear. In the keratoconus group, IOP rose from 13.58 ± 3.09 mmHg to 14.52 ± 2.70 mmHg, while in the postkeratoplasty group, it increased from 12.96 ± 3.16 mmHg to 14.28 ± 2.94 mmHg (*p* < 0.001 for both). In the ocular trauma group, IOP grew from 14.27 ± 2.91 mmHg to 15.97 ± 2.98 mmHg, and in the dry eye disease group, from 14.34 ± 3.12 mmHg to 15.80 ± 3.11 mmHg. Both increases were statistically significant (*p* < 0.01 and *p* < 0.001, respectively, Figure 1(c)).

Following scleral lens wear, all groups exhibited an increase in CCT. After 4 hours of lens wear, the CCT in the keratoconus group increased from 491.08 ± 47.42 μm to 501.70 ± 42.05 μm, and in the postkeratoplasty group from 562.79 ± 115.99 μm to 586.64 ± 97.78 μm. In the ocular trauma and dry eye disease groups, CCT increased from 585.16 ± 57.63 and 501.87 ± 65.51 μm to 596.85 ± 58.82 and 509.49 ± 67.68 μm, respectively. Postwear CCT increased significantly relative to baseline in all four groups, including keratoconus (*p* < 0.001), corneal transplantation (*p* = 0.004), trauma (*p* < 0.001), and dry eye (*p* < 0.001, Figure 1(d)).

**FIGURE 2:**
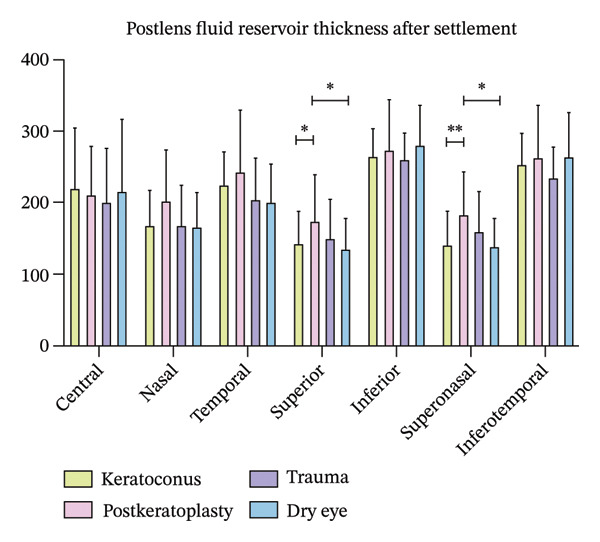
No significant intergroup differences were observed at the central, inferior, nasal, temporal, or inferotemporal locations. However, the postkeratoplasty group demonstrated significantly greater tear reservoir thickness at the superior and superonasal positions compared to the keratoconus and dry eye disease groups (^∗^ *p* < 0.05 and ^∗∗^ *p* < 0.01).

Table 3 presents the tear reservoir thickness at seven measurement locations (central, superior, inferior, nasal, temporal, superonasal, and inferotemporal) before and after lens settling in the four groups. No significant differences in tear reservoir thickness were observed among the four groups at the central, inferior, nasal, temporal, and inferotemporal locations; however, at the superior and superonasal locations, the postkeratoplasty group (174.85 ± 63.78 μm and 183.21 ± 60.07 μm, respectively) exhibited significantly greater tear reservoir thickness compared to the keratoconus (142.36 ± 45.78 μm and 141.82 ± 46.53 μm, respectively) and dry eye (136.35 ± 41.69 μm and 139.42 ± 38.95 μm, respectively) groups, which may be attributable to irregular astigmatism following corneal transplantation (Figure 2).

**TABLE 3:** Postlens tear reservoir thickness at seven points (before and after settlement, μm).

Group		Keratoconus	Postkeratoplasty	Trauma	Dry eye
Central	Before	327.40 ± 98.32	325.35 ± 78.83	310.07 ± 91.48	319.18 ± 94.37
	After	220.18 ± 84.63	211.36 ± 68.12	201.41 ± 74.66	215.79 ± 101.60

Nasal	Before	208.25 ± 47.12	251.62 ± 83.07	208.74 ± 55.50	216.08 ± 53.66
	After	167.97 ± 49.72	202.63 ± 71.49	168.45 ± 55.45	166.58 ± 47.33

Temporal	Before	269.13 ± 48.12	300.48 ± 63.59	263.01 ± 52.90	255.48 ± 49.62
	After	224.67 ± 46.03	243.82 ± 85.55	205.19 ± 57.62	200.88 ± 53.94

Superior	Before	180.72 ± 53.62	216.79 ± 55.88	201.47 ± 69.73	188.85 ± 56.79
	After	142.36 ± 45.78	174.85 ± 63.78	151.22 ± 54.26	136.35 ± 41.69

Inferior	Before	296.04 ± 32.04	336.18 ± 83.57	316.08 ± 57.43	335.59 ± 76.72
	After	265.38 ± 38.38	273.95 ± 70.30	260.42 ± 37.19	280.56 ± 55.70

Superonasal	Before	168.94 ± 53.83	220.52 ± 54.09	204.45 ± 56.79	174.45 ± 40.51
	After	141.82 ± 46.53	183.21 ± 60.07	160.36 ± 55.58	139.42 ± 38.95

Inferotemporal	Before	295.59 ± 38.87	314.40 ± 75.91	295.43 ± 50.68	313.15 ± 62.41
	After	253.89 ± 44.03	263.53 ± 73.47	234.63 ± 43.35	264.28 ± 61.71

### 3.1. Postscleral Lens Wear Questionnaire Follow‐Up

**FIGURE 3:**
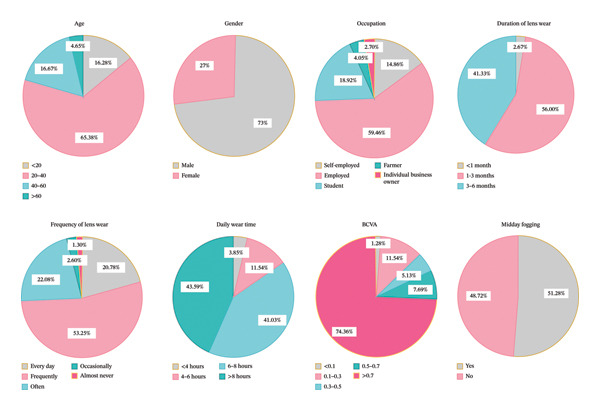
Distribution of questionnaire‐based clinical and demographic parameters among patients wearing scleral lenses. The figure summarizes patient demographics (age and sex), occupational status, and lens‐wearing patterns, including duration of use, wearing frequency, and average daily wearing time. Clinical parameters include BCVA under lens wear and the presence of midday fogging. Data are presented to illustrate the overall distribution and variability of patient‐reported outcomes and usage behaviors across the study cohort.

A total of 79 patients were followed up on this study. The majority of these patients (65.38%) were between 20 and 40 years old, and 73% were male. 4.05% were farmers, 14.86% were self‐employed, 59.46% were employed, and 18.92% were students. As for the duration of scleral lens wear, 56% of the patients had worn lenses for 1–3 months and 41.33% for 3–6 months. A total of 20.78% wore the lenses daily, 53.25% wore them frequently (at least 3 weeks per month), 22.08% wore them often (at least 2 weeks per month), and 2.60% wore them occasionally (at least 1 week per month). The patients had varying daily wear time: 3.85% wore lenses for less than 4 h per day, 11.54% for 4–6 h, 41.03% for 6–8 h, and 43.59% for more than 8 h daily. In terms of BCVA while wearing scleral lenses, 74.36% had BCVA greater than 0.7. In addition, 51.28% of patients reported experiencing “midday fogging” during lens wear (Figure 3).

### 3.2. Subjective Acceptability Following Scleral Lens Wear

**FIGURE 4:**
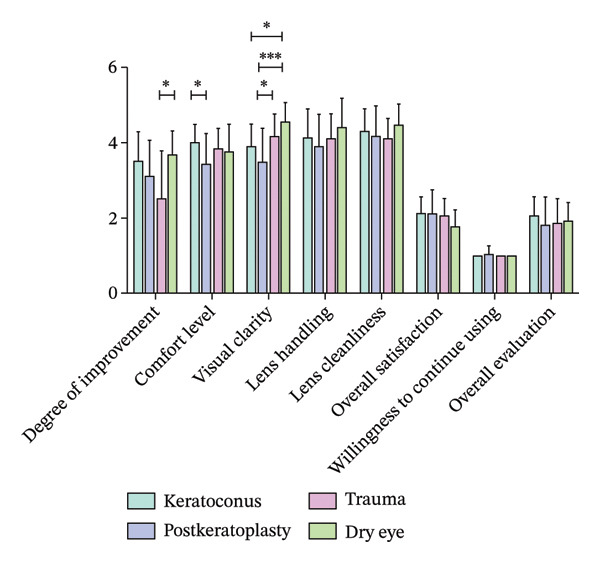
The quality‐of‐life improvement in the trauma group was significantly lower compared to the dry eye disease group (^∗^ *p* < 0.05). The postkeratoplasty group reported significantly lower comfort than the keratoconus group (^∗^ *p* < 0.05). Similarly, the postkeratoplasty group demonstrated significantly lower visual clarity scores compared to both the trauma and dry eye disease groups (^∗^ *p* < 0.05 and ^∗∗^ *p* < 0.001, respectively). Patient ratings for overall satisfaction, lens handling, and cleanliness were consistently high across all four groups, with no significant intergroup differences.

Following scleral lens wear, the degree of improvement in quality of life in the trauma group was significantly lower compared to the dry eye disease group (*p* < 0.05). The postkeratoplasty group reported significantly lower comfort scores compared to the keratoconus group, with the difference being statistically significant (*p* < 0.05). The postkeratoplasty group demonstrated significantly lower scores for visual clarity, compared to the trauma and dry eye disease groups, with the difference being statistically significant (*p* < 0.05 and *p* < 0.001, respectively). The absence of significant corneal astigmatism caused the dry eye disease group to demonstrate the best visual clarity; however, no statistically significant differences among the four groups in subjective scores for lens usability, lens cleanliness, overall satisfaction, willingness to continue using scleral lenses, and overall evaluation were noted (Figure 4).

## 4. Discussion

This study is novel in directly comparing scleral lens outcomes across keratoconus, postkeratoplasty, trauma‐related corneal irregularity, and dry eye disease. This cross‐etiology analysis delineates disease‐specific differences and provides evidence to guide individualized fitting and clinical decision‐making. Scleral lens wear led to a significant improvement in BCVA in all groups, with the greatest gains observed in the keratoconus and dry eye disease groups. In addition, OSDI scores significantly decreased after lens wear, indicating a substantial reduction in ocular discomfort. The majority of patients demonstrated good compliance, with most wearing the lenses for more than 6 hours per day and reporting high levels of comfort and satisfaction. However, a considerable proportion of patients experienced “midday fogging,” suggesting that further optimization in lens care and tear film management may be warranted.

The improvements in visual acuity observed across the four groups highlight the capacity of scleral lenses to correct a wide range of corneal irregularities and optical aberrations. The significant increase in BCVA in the keratoconus group from 0.52 ± 0.14 to 0.94 ± 0.16 confirms previous studies’ findings that scleral lenses can effectively neutralize the irregular astigmatism and higher‐order aberrations associated with ectatic corneal disorders by creating a smooth, regular refractive surface [1, 13]. Similarly, in patients with dry eye disease, the improvement in BCVA (from 0.76 ± 0.23 to 1.03 ± 0.15) may be attributed not only to enhanced optical correction but also to the protective, tear‐filled reservoir provided by the lens, which stabilizes the tear film and reduces surface irregularity and dryness‐induced visual fluctuations [10, 15].

Although significant visual gains were also noted in the postkeratoplasty group (BCVA improved from 0.24 ± 0.19 to 0.57 ± 0.36), the magnitude of this improvement was less pronounced than it was for other groups. This may be related to underlying graft irregularities, residual stromal scarring, or reduced endothelial function, which can limit the extent of visual rehabilitation achievable with a scleral lens [16]. Notably, the ocular trauma group also exhibited marked improvement (from 0.33 ± 0.15 to 0.77 ± 0.20), underscoring the utility of scleral lenses in cases of posttraumatic corneal distortion or scarring. These findings are consistent with those of prior studies, demonstrating the versatility of scleral lenses in managing irregular astigmatism from diverse etiologies [17]. Overall, our results reinforce the role of scleral lenses as a valuable nonsurgical option for visual rehabilitation in complex ocular surface conditions.

This study also demonstrated a significant reduction in ocular surface symptoms across all groups, as reflected by the marked decrease in OSDI scores following scleral lens wear. In particular, the keratoconus and dry eye disease groups exhibited the most pronounced improvements. The tear reservoir beneath the scleral lens protects the cornea from external environmental insults, minimizes friction from blinking, and helps stabilize the tear film, thereby reducing symptom burden [1, 18]. The dry eye disease group, despite relatively good baseline visual acuity, showed significant symptomatic relief after lens wear, which is consistent with prior studies indicating that scleral lenses are highly effective in managing severe ocular surface disease, especially when conventional therapies have failed [3, 19, 20]. Notably, scleral lenses in treating dry eye disease have been shown to reduce both evaporative and aqueous‐deficient symptoms by creating a continuously hydrated environment for the ocular surface [21, 22]. This mechanism also helps explain the significant increase in patients’ comfort and quality of life reported in the follow‐up questionnaire.

An increase in CCT was observed across all groups following 4 hours of scleral lens wear. The magnitude of thickening ranged from approximately 1.5% to 4.2%, which falls within the range of mild, transient corneal edema previously reported during short‐term scleral lens wear. This finding may be attributed to two primary factors: transient corneal edema and the accumulation of postlens tear reservoir [23, 24]. Although modern scleral lenses are fabricated from high oxygen‐permeable materials, their relatively thick profile and limited tear exchange may modestly reduce oxygen transmission to the central cornea, particularly during continuous wear. However, the observed changes were consistent with physiologic, reversible edema described in prior studies [25–27]. No clinical signs of hypoxia‐related compromise were detected, supporting the overall short‐term safety of scleral lens wear in this cohort.

A statistically significant increase in IOP was observed after scleral lens wear in all groups; however, the mean elevation was less than 2 mmHg and remained within the normal physiological range, suggesting limited short‐term clinical impact. Concurrent increases in CCT may have contributed to the overestimation of IOP, as CCT‐adjusted values were not calculated. In addition, scleral lens haptic compression may elevate episcleral venous pressure and transiently reduce aqueous outflow, as previously described [28]. Although no acute IOP‐related adverse events were observed, IOP monitoring may be warranted in patients at high risk for glaucoma, and longitudinal studies are required to establish long‐term safety.

Our analysis of postlens tear reservoir thickness across seven measurement locations after lens settling revealed that most positions (central, inferior, nasal, temporal, and inferotemporal) indicated no significant intergroup differences, suggesting that scleral lenses generally provide a stable and uniform postlens tear film across the majority of the ocular surface. However, the postkeratoplasty group exhibited significantly greater tear reservoir thickness at the superior and superonasal locations compared to the other groups. This observation aligns with evidence that corneal grafts frequently induce irregular astigmatism and asymmetrical corneal elevation, particularly in the superior region where surgical incisions and sutures are commonly placed [29]. This underscores the importance of carefully evaluating superior fitting zones in postkeratoplasty patients, as excessive clearance may predispose patients to increased midday fogging and reduced lens stability, potentially compromising visual outcomes and comfort.

Interestingly, although all groups reported high levels of subjective comfort and visual clarity, the postkeratoplasty group exhibited relatively lower comfort and visual clarity scores. This could have been the result of persistent irregularities on the graft–host junction, localized elevations at the suture sites, or reduced corneal sensitivity, which may alter the subjective experience of lens wear [30]. These findings underscore the importance of careful fitting and ongoing follow‐up in patients with complex corneal morphology to ensure optimal lens alignment and tear exchange.

The patient‐reported outcomes derived from the follow‐up questionnaire further highlight the overall effectiveness and acceptability of scleral lenses in managing ocular surface disease. Most patients reported high levels of comfort, ease of lens handling, and improved quality of life, with approximately 75% achieving a postwear BCVA ≥ 0.7 in daily wear. Furthermore, most participants wore their lenses frequently or daily, with more than 40% wearing them for over 8 h per day, an indication of good tolerance and integration into daily life.

Despite their overall satisfaction with the lenses, nearly half of the patients reported experiencing “midday fogging,” a phenomenon attributed to the accumulation of debris or mucin within the postlens tear reservoir over time. The prevalence of midday fogging in this study was similar to previously reported rates [31, 32]. Specifically, postkeratoplasty eyes demonstrated significantly greater tear reservoir thickness at the superior and superonasal locations, regions where graft–host junction irregularity and suture‐related elevation are commonly present. Excessive regional clearance in these areas may predispose to tear stagnation and debris accumulation, thereby increasing the likelihood of midday fogging.

These findings suggest that beyond central clearance optimization, attention to regional asymmetry in tear reservoir thickness, particularly in postkeratoplasty eyes, may be critical for minimizing fogging. Adjustments such as reducing excessive vault in elevated regions, improving scleral alignment to facilitate tear exchange, and addressing underlying ocular surface inflammation may help mitigate this complication.

This study has several limitations. The relatively small sample size in each subgroup may reduce the generalizability of the findings, and the short follow‐up period limits the assessment of long‐term outcomes. In addition, dynamic fitting parameters such as lens decentration and tear exchange were not quantitatively analyzed. IOP measurements were not adjusted for changes in CCT, which may have influenced the observed IOP elevation. Furthermore, midday fogging was assessed using a binary self‐reported measure (presence vs. absence) without grading its severity. This may underestimate the clinical burden and interindividual variability of this complication. Future studies should include larger cohorts, longer follow‐up, CCT‐adjusted IOP analysis, standardized severity scales for midday fogging, and more advanced imaging approaches to refine fitting strategies and better address persistent challenges associated with scleral lens wear.

In conclusion, scleral lenses yield high patient satisfaction and can provide effective visual rehabilitation across diverse ocular surface diseases, offering sustained improvements in comfort and only occasional reports of midday fogging. By integrating objective and subjective assessments, this study underscores the versatility and safety of scleral lenses while revealing disease‐specific differences in the outcomes of scleral lens treatment. Future work should refine OCT‐guided fitting, address tear reservoir debris, and conduct long‐term multietiology comparisons to optimize results.

## Funding

The authors acknowledge the financial support of the National Natural Science Foundation of China (Project number: 82301161), the Zhejiang Province Traditional Chinese Medicine Science and Technology Plan Project (Project number: 2024ZL199), and the Wenling City Social Development Science and Technology Project (Project numbers: 2023S00168 and 2024S00251).

## Ethics Statement

The study was approved by the Institutional Review Board of the Affiliated Second Hospital of Zhejiang University.

## Conflicts of Interest

The authors declare no conflicts of interest.

## Supporting Information

Additional supporting information can be found online in the Supporting Information section.

## Supporting information


**Supporting Information** Supporting Figure 1: Postfitting questionnaire for scleral lens wear.

## Data Availability

The data that support the findings of this study are available on request from the corresponding author. The data are not publicly available due to privacy or ethical restrictions.
