# Supplementary material for: Comparative Evaluation of Scleral Lens Treatment in Ocular Surface Diseases: A Prospective Study
Source: J Ophthalmol. 2026 May 23;2026:1921583. doi: 10.1155/joph/1921583 (PMC13197798; doi:10.1155/joph/1921583)
Supplement: Supplementary file 1 — Supporting Information Supporting Figure 1: Postfitting questionnaire for scleral lens wear. [file JOPH-2026-1921583-s001.docx]

Post-Fitting Questionnaire for Scleral Lens Wear

Name:

(Please print your name and check the appropriate boxes based on your actual situation.)

1. Your age:

□ <18 □ 18–25 □ 26–35 □ 36–45 □ ≥46

2. Gender:

□ Male □ Female

3. Duration of scleral lens wear:

□ Less than 1 month □ 1–3 months

□ 3–6 months □ More than 6 months

4. Your occupation:

□ Homemaker □ Self-employed □ Employed

□ Student □ Farmer □ Retired

□ Private business owner □ Other (please specify): _____

5. Frequency of scleral lens wear after fitting:

□ Daily wear

□ Frequent wear: Worn for at least 3 weeks per month

□ Regular wear: Worn for at least 2 weeks per month

□ Occasional wear: Worn for at least 1 week per month

□ Rare wear: Worn for less than 1 week per month

6. Average daily wearing time of scleral lenses:

□ ≤4 hours □ 4–6 hours □ 6–8 hours □ >8 hours

7. Best-corrected visual acuity (BCVA) with scleral lenses:

□ <0.1 □ 0.1–0.3 □ 0.3–0.5

□ 0.5–0.7 □ >0.7

8. Midday fogging occurrence during scleral lens wear:

□ Present □ Absent

9. Ocular discomfort during scleral lens wear (e.g., dryness, foreign body sensation, pain, fatigue):

*Symptom score（ frequency）:

(0 = Never; 1 = Occasionally; 2 = Half the time; 3 = Most of the time; 4 = Constantly)

|  | Symptom | 0 | 1 | 2 | 3 | 4 |
| --- | --- | --- | --- | --- | --- | --- |
| A1 | Foreign body sensation |  |  |  |  |  |
| A2 | Ocular dryness |  |  |  |  |  |
| A3 | Aching/pain |  |  |  |  |  |
| A4 | Ocular fatigue |  |  |  |  |  |
| A5 | Heavy eyelid sensation |  |  |  |  |  |
| A6 | Conjunctival redness |  |  |  |  |  |

If symptoms present, rate their severity:

(0 = None; 1 = Mild; 2 = Moderate; 3 = Severe; 4 = Very severe)

|  | Symptom | 0 | 1 | 2 | 3 | 4 |
| --- | --- | --- | --- | --- | --- | --- |
| B1 | Foreign body sensation |  |  |  |  |  |
| B2 | Ocular dryness |  |  |  |  |  |
| B3 | Aching/pain |  |  |  |  |  |
| B4 | Ocular fatigue |  |  |  |  |  |
| B5 | Heavy eyelid sensation |  |  |  |  |  |
| B6 | Conjunctival redness |  |  |  |  |  |

10. Post-fitting improvement in dry eye symptoms and quality of life (QoL):

0 = No improvement (neither dry eye symptoms nor QoL improved)

1 = Minimal improvement (either dry eye symptoms or QoL improved slightly)

2 = Slight improvement (either dry eye symptoms or QoL improved moderately)

3 = Improvement (both dry eye symptoms and QoL improved slightly)

4 = Notable improvement (both dry eye symptoms and QoL improved moderately)

5 = Significant improvement (both dry eye symptoms and QoL improved markedly)

11. How would you rate the comfort level after wearing scleral lenses?

0 = Painful, lenses intolerable

1 = Extremely uncomfortable with significant irritation or intolerance

2 = Mildly uncomfortable with noticeable irritation or intolerance

3 = Comfortable with lens awareness but no irritation

4 = Very comfortable with occasional lens awareness

5 = Excellent, no lens awareness

12. How would you rate your vision after wearing scleral lenses?

0 = Unacceptable, lenses unwearable

1 = Very poor, unsatisfactory vision

2 = Poor, blurred vision but tolerable

3 = Good, occasional blurred vision but acceptable

4 = Very good, minimal blurred vision and acceptable

5 = Excellent, no visual blur

13. How would you rate the handling of scleral lenses after wear?

0 = Extremely poor, unable to handle lenses

1 = Very poor, significant difficulty with rare successful insertion/removal

2 = Poor, occasional difficulty with insertion/removal

3 = Good, minor issues but generally successful insertion/removal

4 = Very good, occasional handling difficulties

5 = Excellent, no issues with insertion/removal

14. How would you rate the cleanliness of scleral lenses after wear?

0 = Extremely poor, unable to clean lenses

1 = Very poor, mostly ineffective cleaning

2 = Poor, occasional cleaning difficulties

3 = Good, minor issues but generally cleanable

4 = Very good, occasional cleaning challenges

5 = Excellent, lenses remain thoroughly clean

15. What is your overall satisfaction with the scleral lens fitting process?

□ Very satisfied □ Moderately satisfied □ Neutral □ Somewhat dissatisfied

□ Completely dissatisfied

16. Are you willing to continue using scleral lenses? (Single-choice question)

□ Yes □ No

17. What is your overall evaluation of scleral lenses?

□ Excellent □ Good □ Neutral □ Poor □ Very poor
